# The underlying mechanisms for development of hypertension in the metabolic syndrome

**DOI:** 10.1186/1475-2891-7-10

**Published:** 2008-04-17

**Authors:** Hidekatsu Yanai, Yoshiharu Tomono, Kumie Ito, Nobuyuki Furutani, Hiroshi Yoshida, Norio Tada

**Affiliations:** 1 Department of Internal Medicine, The Jikei University School of Medicine, Chiba, Japan; 2 Department of Laboratory Medicine, The Jikei University School of Medicine, Chiba, Japan

## Abstract

High blood pressure is an important constituent of the metabolic syndrome. However, the underlying mechanisms for development of hypertension in the metabolic syndrome are very complicated and remain still obscure. Visceral/central obesity, insulin resistance, sympathetic overactivity, oxidative stress, endothelial dysfunction, activated renin-angiotensin system, increased inflammatory mediators, and obstructive sleep apnea have been suggested to be possible factors to develop hypertension in the metabolic syndrome. Here, we will discuss how these factors influence on development of hypertension in the metabolic syndrome.

## Introduction

The metabolic syndrome is characterized by the simultaneous occurrence of metabolic abnormalities including obesity, glucose intolerance, dyslipidemia, and hypertension, that result in a marked increase in cardiovascular morbidity and mortality [1-3]. High blood pressure is a classical feature of the metabolic syndrome, and it has been reported that the metabolic syndrome is present in up to one third of hypertensive patients [4,5]. Therefore, high blood pressure is included in the definition for the metabolic syndrome that presented by the World Health Organization, the National Cholesterol Education Program, the International Diabetes Federation, and the American Heart Association/National Heart, Lung, and Blood Institutes [1-3,6]. Blood pressure levels are strongly associated with visceral obesity and insulin resistance [7], which are the main pathophysiologic features underlying the metabolic syndrome. Here, we will discuss the underlying mechanisms for development of hypertension in the metabolic syndrome.

### The underlying mechanisms for development of hypertension in the metabolic syndrome

**Figure 1:**
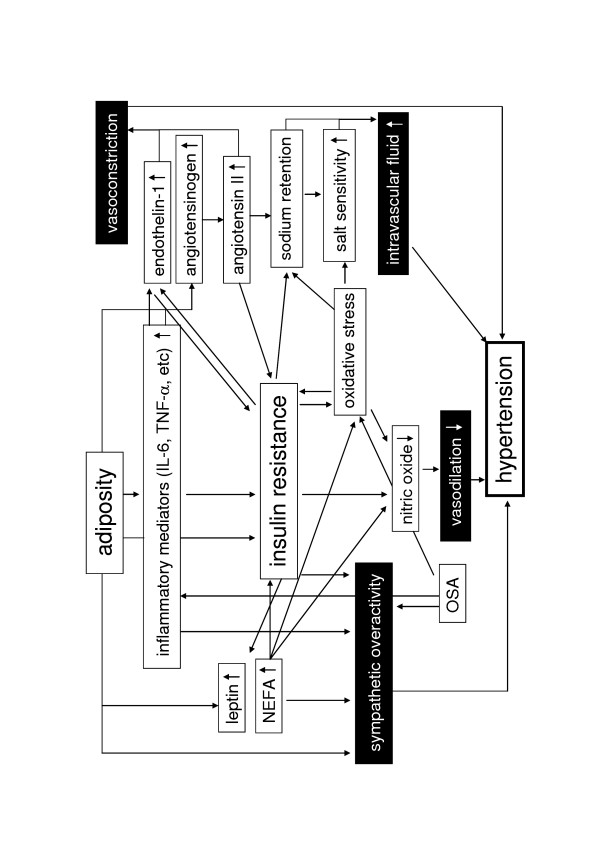
**Proposed mechanisms for development of hypertension in the metabolic syndrome.** IL-6, interleukin-6; NEFA, non-esterified fatty acids; TNF-α, tumor necrosis factor-α; OSA, obstructive sleep apnea.

Proposed mechanisms for development of hypertension in the metabolic syndrome were shown in Figure 1.

#### Visceral obesity

Excess food intake and physical inactivity underlie the growing worldwide epidemic of obesity. Hyperglycemia, hyperlipidemia, and hypertension are common in obese individuals [8,9]. Visceral obesity has been suggested to play a fundamental role in the simultaneous development of these disorders [10]. Recent studies have demonstrated that adipose tissue is a major endocrine organ that secrets a variety of bioactive substances, termed adipocytokines. Adipocytokines secretion are altered as obesity develops, which may induce the metabolic disorders. As shown in Figure 1, accumulated visceral adipose tissue produce and secrete a number of adipocytokines, such as leptin, tumor necrosis factor-α (TNF-α), interleukin-6 (IL-6), angiotensinogen, and non-esterified fatty acids (NEFA), which induce development of hypertension [11]. Visceral obesity is the main cause of the metabolic syndrome, and is associated with development of hypertension in the metabolic syndrome via a variety of pathways (Figure 1).

#### Insulin resistance

Insulin resistance is the main pathophysiologic feature of the metabolic syndrome. Several mechanisms connect insulin resistance with hypertension in the metabolic syndrome. An anti-natriuretic effect of insulin has been established by accumulating data indicating that insulin stimulates renal sodium re-absorption [12-14]. This anti-natriuretic effect is preserved, and may be increased in individuals with insulin resistance, and this effect may play an important role for development of hypertension in the metabolic syndrome [15]. Strazzullo P, et al. investigated the relationship between the metabolic syndrome and renal tubular sodium handling [16]. In their study, proximal fractional sodium re-absorption (FPRNa) was significantly greater in individuals with the metabolic syndrome, as compared with those without the metabolic syndrome [16]. Further, in untreated obese individuals, age-adjusted FPRNa was significantly greater in individuals with insulin resistance as compared with those without insulin resistance [16]. Insulin resistance is also associated with development of salt-sensitive hypertension through the anti-natriuretic effect of insulin [17].

In vitro studies have shown that insulin stimulates both endothelin-1 production and its action on the vascular wall [18]. In vivo study has also demonstrated that high serum insulin level is associated with an increase in circulating endothelin-1 in healthy and insulin-resistant individuals [18]. Endothelin-1 receptor antagonism effectively reduced blood pressure in animal models of insulin resistance and hypertension [18], suggesting a significance of endothelin-1 in the pathogenesis of hypertension in insulin resistance.

#### Sympathetic overactivity

Serum catecholamine concentrations and muscle sympathetic nervous activity (MSNA) were significantly increased in obese individuals as compared with lean individuals [19], and MSNA in subjects with central obesity were significantly greater than those in individuals with peripheral obesity [19]. Elevated resting heat rates [20,21], and baroreflex dysfunction have been reported to play an important role in development of hypertension in the metabolic syndrome [22]. Individuals with obstructive sleep apnea (OSA) have a high prevalence of the metabolic syndrome [23,24], and OSA has been reported to be associated with sympathetic overactivity. Obese individuals exhibit an activated renin-angiotensin system [25], which induces hypertension. The renin-angiotensin system and sympathetic nervous system are linked by a positive feedback relationship [26]. Insulin resistance, increased leptin and NEFA levels have been indicated to be possible factors augmenting sympathetic nervous activation in the metabolic syndrome [27]. NEFA has been reported to raise blood pressure, heart rate, and α1-adrenoceptor vasoreactivity, while reducing baroreflex sensitivity, endothelium-dependent vasodilatation, and vascular compliance [28]. Insulin resistance increases plasma leptin levels, and leptin has been reported to elevate sympathetic nervous activity, suggesting that leptin-dependent sympathetic nervous activation may contribute to an obesity-associated hypertension [29]. Accumulating data suggest that metabolic syndrome is associated with markers of adrenergic overdrive [30].

#### Oxidative stress and endothelial dysfunction

In rats with the metabolic syndrome, induced by chronic consumption of a high fat, high refined sugar [31], hypertension is associated with oxidative stress [32], avid nitric oxide (NO) inactivation, and down-regulation of NO synthase (NOS) isoforms and endothelial NOS activator [32], suggesting that oxidative stress and endothelial dysfunction may be strongly associated with development of hypertension in the metabolic syndrome. Further, recent evidences suggest that oxidative stress, which is elevated in the metabolic syndrome [33], is associated with sodium retention and salt sensitivity [34].

In non-diabetic human subjects, lipid peroxidation, represented by plasma thiobarbituric acid reactive substance and urinary 8-epi-prostaglandin-F2α, were significantly and positively correlated with body mass index and waist circumference, indicating that fat accumulation is correlated with oxidative stress in humans [33]. Cross-sectional data from 2,002 non-diabetic subjects of the community-based Framingham Offspring Study has shown that systemic oxidative stress is associated with insulin resistance [35]. Insulin resistance induces an impairment in phosphatidylinositol 3-kinase (PI3K) – dependent signaling, which in endothelium may cause imbalance between production of nitric oxide and secretion of endothelin-1, leading to endothelial dysfunction [36]. Epidemiological studies strongly support a reciprocal relationship between endothelial dysfunction, which contributes to development of hypertension, and insulin resistance [36]. In a prospective cohort study, each one-unit decrease of flow-mediated dilatation was associated with a significant 16% (95% confidence interval: 12–33%) increase in the multiple-adjusted relative risk of incident hypertension, suggesting that an impaired endothelial vasomotor function precedes and predicts the future development of hypertension [37].

#### Activated renin-angiotensin system

The renin-angiotensin system (RAS) plays a crucial role in blood pressure regulation, by affecting renal function and by modulating vascular tone. The activity of the RAS appears to be regulated by food intake, and overfeeding of rodents has been reported to lead to increased formation of angiotensin II in adipocytes [38]. Angiotensinogen, angiotensin converting enzyme, and type 1 angiotensin receptor gene are widely expressed in human adipose tissue [39], and production of angiotensin II and angiotensinogen in adipose tissue may be increased in obese subjects. Goodfriend TL, et al. measured plasma aldosterone levels in adults with various values of body mass index [40]. Plasma aldosterone level was higher in obese subjects, but could not be explained by renin and potassium [40]. The best predictor for plasma aldosterone level was abdominal obesity [40]. Elevated renin and aldosterone levels have been observed in subjects with multiple risk factors as compared with those without multiple risk factors [41]. Plasma aldosterone has been reported to be significantly associated with the metabolic syndrome and also with obesity-related hypertension [42,43].

There are accumulating data indicating that angiotensin II inhibits the action of insulin via angiotensin type 1 receptor, in vascular muscle tissue, by interfering with insulin signaling through PI3K and its downstream protein kinase B (Akt) signaling pathway [44]. This inhibitory action of angiotensin II is mediated through stimulation of RhoA activity and oxidative stress [44]. Increased RhoA activity and reactive oxygen species inhibit PI3K/Akt signaling, resulting in decreased NO production in endothelial cell and increased vasoconstriction [44]. Activated RAS may contribute to development of hypertension in the metabolic syndrome.

#### Increased inflammatory mediators

Recent cohort studies have demonstrated that high-sensitivity C-reactive protein (hsCRP) independently presents additive prognostic values at all levels of metabolic syndrome [45]. Ridker PM, et al. suggest a consideration of adding hsCRP as a clinical criterion for metabolic syndrome [45]. Abnormalities in inflammatory mediators have been also reported to be implicated with development of hypertension. A positive relationship between increased serum levels of CRP and the risk for development of incident hypertension in participants of the Women's Health Study [46]. Grundy SM suggests a significant association among inflammation, hypertension, and the metabolic syndrome [47].

TNF-α is involved in the pathophysiology of hypertension in the metabolic syndrome. TNF-α stimulates the production of endothelin-1 and angiotensinogen [48,49]. The TNF-α gene locus seems to be involved in human insulin resistance-mediated hypertension [50]. Serum TNF-α concentration has been reported to be positively correlated with systolic blood pressure and insulin resistance in humans [51], and increased TNF-α secretion has been observed in monocytes from hypertensive patients [52].

IL-6 is a multifunctional cytokine which mediates inflammatory responses. Recent study demonstrated that blood pressure was a significant and independent predictor of serum IL-6 concentrations in women [53]. IL-6 stimulates the central nervous system and sympathetic nervous system, which may result in hypertension [54,55]. The administration of IL-6 leads to elevation in heart rate and serum norepinephrine levels in women [56]. Further, IL-6 induces an increase in plasma angiotensinogen and angiotensin II [57], leading to development of hypertension.

#### Obstructive sleep apnea

Of 146 patients with OSA, 88 (60%) had the metabolic syndrome, whereas 33 of 82 patients (40%) without OSA had the metabolic syndrome, suggesting high prevalence of the metabolic syndrome in OSA patients [58]. The proportion with hypertension was significantly higher in the OSA group (77%) than in the non-OSA group (51%), indicating a significant association between OSA and hypertension [58]. Kono M et al. also demonstrated that the percentage of hypertensive patients were significantly higher in the OSA group (45%) than in the non-OSA group (15%) [59]. There are accumulating evidences suggesting a significant association between the metabolic syndrome and OSA or its components [60]. Patients with OSA are often considered to be obese, however, Kono M et al. reported that OSA was associated with hypertension, dyslipidemia, and hyperglycemia, independent of visceral obesity [59]. Recent epidemiological and clinical data suggest a crucial role of OSA in development of hypertension, however, associations of OSA with insulin resistance and dyslipidemia are controversial [60]. Visceral obesity remains a confounding issue in studying the association between OSA and the metabolic syndrome.

OSA is characterized by an increased number of sympathetic bursts, a raised plasma norepinephrine concentration, and a reduction in baroreflex sensitivity [23,24,61], which leaves no doubt as to the existence of sympathetic activation induced by baroreflex dysfunction, much like what has been observed in the metabolic syndrome. In OSA, the nocturnal episodes of hypoxia and hypercapnia induce the stimulation of arterial chemoreceptors, which could induce sympathostimulating effects [61]. Hyperleptinemia, insulin resistance, elevated angiotensin II and aldosterone levels, oxidative stress, inflammation, and endothelial dysfunction have been also suggested to be possible mechanisms whereby OSA may contribute to development of hypertension [62].

## Conclusion

Visceral obesity, insulin resistance, oxidative stress, endothelial dysfunction, activated renin-angiotensin system, increased inflammatory mediators, and obstructive sleep apnea have been proposed to be possible factors to develop hypertension in the metabolic syndrome. These factors may induce sympathetic overactivity, vasoconstriction, increased intravascular fluid, and decreased vasodilatation, leading to development of hypertension in the metabolic syndrome.
